# Differences in PLA_2_ Constitution Distinguish the Venom of Two Endemic Brazilian Mountain Lanceheads, *Bothrops cotiara* and *Bothrops fonsecai*

**DOI:** 10.3390/toxins14040237

**Published:** 2022-03-25

**Authors:** Pedro G. Nachtigall, Luciana A. Freitas-de-Sousa, Andrew J. Mason, Ana M. Moura-da-Silva, Felipe G. Grazziotin, Inácio L. M. Junqueira-de-Azevedo

**Affiliations:** 1 Laboratório de Toxinologia Aplicada, Center of Toxins, Immune-Response and Cell Signaling (CeTICS), Instituto Butantan, São Paulo, SP 05503-900, Brazil; 2 Laboratório de Imunopatologia, Instituto Butantan, São Paulo, SP 05503-900, Brazil; luciana.sousa@esib.butantan.gov.br (L.A.F.-d.-S.); ana.moura@butantan.gov.br (A.M.M.-d.-S.); 3 Department of Evolution, Ecology, and Organismal Biology, The Ohio State University, Columbus, OH 43210, USA; mason.501@osu.edu; 4 Laboratório de Coleções Zoológicas, Instituto Butantan, São Paulo, SP 05503-900, Brazil; felipe.grazziotin@butantan.gov.br

**Keywords:** transcriptome, proteome, venom, evolution, gene expression

## Abstract

Interspecific differences in snake venom compositions can result from distinct regulatory mechanisms acting in each species. However, comparative analyses focusing on identifying regulatory elements and patterns that led to distinct venom composition are still scarce. Among venomous snakes, *Bothrops cotiara* and *Bothrops fonsecai* represent ideal models to complement our understanding of the regulatory mechanisms of venom production. These recently diverged species share a similar specialized diet, habitat, and natural history, but each presents a distinct venom phenotype. Here, we integrated data from the venom gland transcriptome and miRNome and the venom proteome of *B. fonsecai* and *B. cotiara* to better understand the regulatory mechanisms that may be acting to produce differing venom compositions. We detected not only the presence of similar toxin isoforms in both species but also distinct expression profiles of phospholipases A${}_{2}$ (PLA2) and some snake venom metalloproteinases (SVMPs) and snake venom serine proteinases (SVSPs) isoforms. We found evidence of modular expression regulation of several toxin isoforms implicated in venom divergence and observed correlated expression of several transcription factors. We did not find strong evidence for miRNAs shaping interspecific divergence of the venom phenotypes, but we identified a subset of toxin isoforms whose final expression may be fine-tuned by specific miRNAs. Sequence analysis on orthologous toxins showed a high rate of substitutions between PLA2s, which indicates that these toxins may be under strong positive selection or represent paralogous toxins in these species. Our results support other recent studies in suggesting that gene regulation is a principal mode of venom evolution across recent timescales, especially among species with conserved ecotypes.

## 1. Introduction

Snakes compose a monophyletic group that represents one of the most successful vertebrate radiations, comprising more than 3900 species inhabiting almost all continents [1,2,3]. The evolutionary processes that shaped such massive diversity included the development of the venom production and delivery system, which define the Caenophidia group known as Endoglyptodonta [4]. This group of advanced snakes has the ability to produce, secrete, and inject venom that aids in subduing prey and defending against predators [5]. Among Endoglyptodonta, the family Viperidae evolved highly complex arsenals of venom and specialized fangs that allow easy injection of these toxins.

Within Viperidae, the genus *Bothrops* (lanceheads) is highly diverse and is widely distributed across Central and South America [6]. The diversity of *Bothrops* is frequently classified in six monophyletic species groups that are characterized by their distinct morphology and natural history [7,8]. Most species of *Bothrops* are diet generalists, preying on small mammals, squamates, amphibians, birds, and even invertebrates. However, species included in the *Bothrops alternatus* group are diet specialists [9]. *Bothrops ammodytoides*, *B. alternatus*, *B. cotiara*, and *B. fonsecai* prey mainly upon rodents while *B. itapetiningae* represents the generalist exception in this group [9]. The existence of a monophyletic group composed mainly of diet specialists within a snake genus characterized by diet generalists makes the *B. alternatus* group an ideal system to understand the evolutionary context of interspecific differences in venom composition.

Within the *B. alternatus* group, *B. cotiara*, and *B. fonsecai* are distributed on disjunct highland areas of south and southeastern Brazil [10], mainly in regions that combine montane grasslands and Araucaria moist forest (subtropical broadleaf forest characterized by the presence of *Araucaria angustifolia*). *B. cotiara* also extends its distribution to lowlands in the relict patches of Brazilian Atlantic Forest in northeastern Argentina [10]. These two species are sister groups that diverged around 4 Mya [11], but maintained extremely similar external morphology and aspects of their natural history [12]. Despite their similar biological traits, a previous venom proteomics study revealed that *B. fonsecai* presents a high abundance of phospholipase A${}_{2}$ (PLA2) in its venom, whereas *B. cotiara* has an absence of PLA2 [13]. However, a recent study revealed the presence of PLA2 in the venom proteome of a population of *B. cotiara* from Misiones, Argentina [14], suggesting that *B. cotiara* may exhibit higher intraspecific variation than previously reported. Despite observed differences in venom composition, comparisons of venom function from both species reveal a similar level of toxicity and lethality [15]. Together, these data indicate that both species shared an ancestral venom phenotype and that differences in the venom composition had a limited functional impact. In this context, *B. fonsecai* and *B. cotiara* represent an interesting model to test the roles of different molecular mechanisms in generating venom variation.

Variations in venom components are commonly observed, and such variation can be extensive at both inter- and intraspecific levels [16,17,18]. Patterns of variation in venom composition may be associated with the expression of specific toxins in regulatory submodules, suggesting that modular expression regulation may be a key mechanism for venom evolution [19,20]. Venom variation can also occur ontogenetically between juveniles and adults [21,22]. Furthermore, some studies have shown that miRNAs may modulate this ontogenetic shifting in the venom composition of rattlesnake [23,24] and sea snake species [25]. These studies indicate that posttranscriptional regulation may be a source of regulatory mechanisms that are likely acting on the evolution and maintenance of distinct venom phenotypes.

Here, we investigated and compared the venom gland transcriptome, proteome, and miRNome of *B. fonsecai* and *B. cotiara* to better understand the mechanisms involved in the evolution of venom composition in both species. We characterized the venom components of representative individuals of each species, which allowed us to identify variations within and between species. We performed coexpression analysis to identify modules of variation that may be related to the transcriptional regulation of venom production and also identified miRNAs that may be related to the posttranscriptional regulation of specific toxins. We examined putative gain and loss events of toxin genes between these closely related species. Additionally, we performed enzymatic activity assays to functionally validate the proteomic data observed in each species.

## 2. Results and Discussion

### 2.1. Venom Characterization and Comparative Analysis

**Figure 1:**
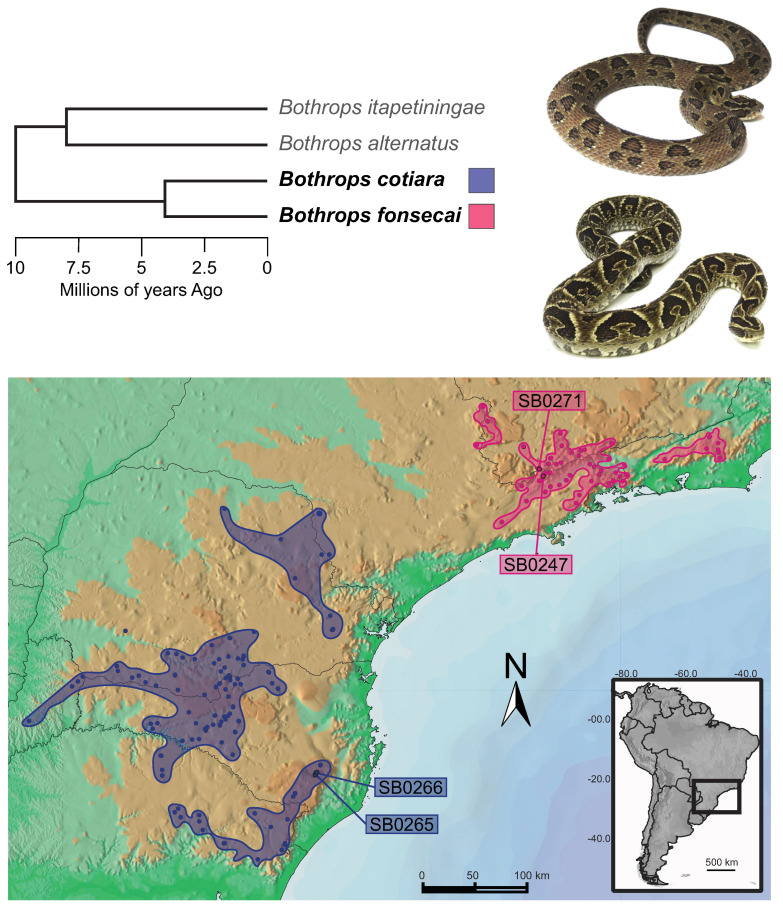
Phylogeny and divergence time of *Bothrops* species from the *B. alternatus* group [11,29] and distribution map for *B. fonsecai* and *B. cotiara* [10]. The map shows part of the South and part of the Southeast regions of Brazil. Sampled localities are shown as dots. The specimens of *B. cotiara* were collected near Urubici city (SB0265 and SB0266). One specimen of *B. fonsecai* was collected near Campos do Jordão city (SB0247), whereas the other specimen was collected near São Bento do Sapucaí city (SB0271).

To examine the evolutionary mechanisms underlying venom divergence we sequenced, assembled, and characterized the venom gland transcriptomes and proteomes of two specimens of *B. cotiara* and two specimens of *B. fonsecai* obtained from similar localities within the distribution ranges of each species (Figure 1). The number of recovered toxin transcripts and toxin families was consistent with those of other *Bothrops* venom gland transcriptomes [22,26,27,28].

**Figure 2:**
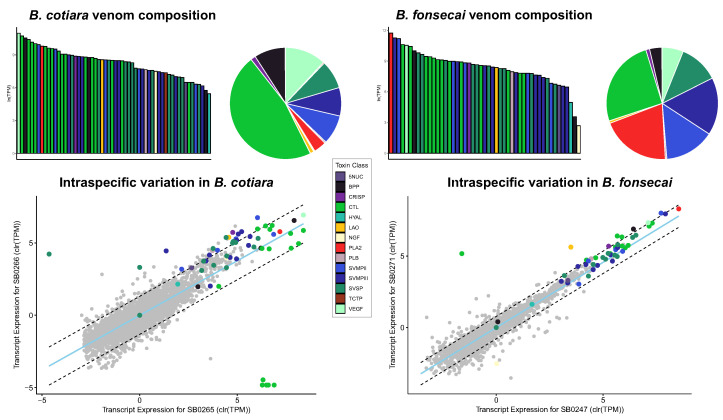
Venom gland transcriptomes of *Bothrops cotiara* (at the **left**) and *B. fonsecai* (at the **right**). At the (**top**), barplots and pie charts represent the average venom composition of each species classified by toxin class. At the (**bottom**), scatter plots show intraspecific variation in transcription expression. For all plots, the data was centered-log transformed to account for their compositional nature. Dashed lines in scatter plots denote the 99% confidence interval of nontoxin expression and the light blue line shows the line of best fit based on orthogonal residuals. TPM—Transcripts Per Million.

For *B. cotiara*, we recovered 5468 annotated transcripts, including 58 toxins from 14 toxin families (Table S1 in Supplementary Materials). The venom transcriptome of *B. cotiara* was dominated by C-type lectin (CTL) expression, which accounted for 46.7% of toxin expression (Figure 2; Figure S1 in Supplementary Materials). Snake venom metalloproteinases (SVMPs), snake venom vascular endothelial growth factor (VEGF-F), Bradykinin Potentiating Peptides (BPPs), and snake venom serine proteases (SVSPs) were also abundant in the *B. cotiara* venom gland transcriptome, representing 16.7%, 11.8%, 9.0%, and 8.2%, respectively. PLA2 represented only 3.6% of the toxin expression, whereas all other toxin families together accounted for less than 3.7% and represented minor components. Of the total toxins assembled for *B. cotiara*, 24 were expressed outside of the 99th percentile of the nontoxin null distribution in the intraspecific variation analysis (Figure 2). Significant variation was mainly detected for CTLs, SVMPs, and SVSPs; however, we also found expression differences for Cysteine-rich secretory protein (CRISP), L-amino acid oxidase (LAO), and phospholipase B (PLB). This finding indicates that *B. cotiara* may present considerable intraspecific variation in toxin expression. We observed a similarly low level of PLA2 expression in the transcriptome of both individuals, which implicates a presence of a PLA2 gene in *B. cotiara* genome [13].

For *B. fonsecai*, we recovered 6870 annotated transcripts, which included 49 toxins from 13 toxin families (Table S2 in Supplementary Materials). SVMPs, CTL, and PLA2 were highly abundant by representing 31.0%, 25.3%, and 20.1% of toxin expression, respectively. SVSPs and VEGF-F were also abundant accounting for 11.4% and 6.1%, whereas BPP represented only 3.5% of toxin expression (Figure 2; Figure S2 in Supplementary Materials). All other toxin families together accounted for less than 2.3% of toxin expression and represented minor components. In this species, we detected 10 toxin transcripts with expression outside of the 99th percentile of the nontoxin null distribution, indicating a low level of intraspecific variation (Figure 2). These 10 transcripts were spread among the toxin families of BPP, CRISP, CTL, LAO, venom nerve growth factor (NGF), SVMPs, and VEGF-F.

**Figure 3:**
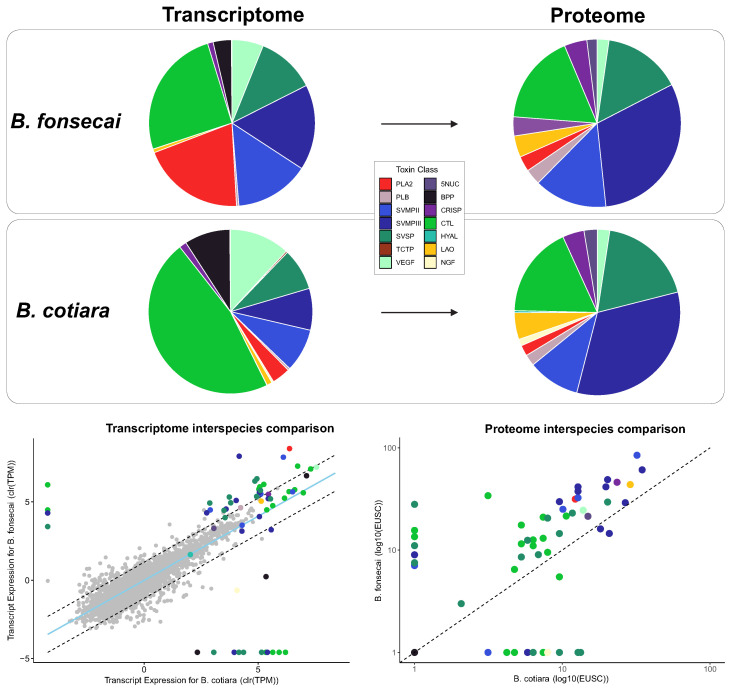
Venom composition of transcriptome and proteome of *Bothrops cotiara* and *B. fonsecai* and interspecific comparisons. Transcriptome data were centered and log transformed to account for their compositional nature, and the dashed lines denote the 99% confidence interval of nontoxin expression while the light blue line is the line of best fit based on orthogonal residuals. In the proteome chart, the dashed line indicates a hypothetical correspondence of protein abundances between species. TPM—Transcripts Per Million. EUSC—Exclusive Unique Spectra Counts.

To perform the interspecific variation analysis, we inferred orthologous transcripts between species using OrthoFinder. We identified 3499 putative ortholog clusters, which included 61 orthologous toxins (Table S3 in Supplementary Materials). The comparative analysis of toxin expression between *B. cotiara* and *B. fonsecai* revealed a set of 40 toxin transcripts with differential expression (Figure 3). Significant variation was detected for PLA2, SVMPs, SVSPs, CTLs, NGF, and BPP. Most striking, the amount of PLA2 in the transcriptome was 6x higher in *B. fonsecai* than in *B. cotiara*. Among SVMPs with interspecific variation, we noticed that eight orthologous isoforms presented higher expression in *B. fonsecai*, whereas only one isoform presented higher expression in *B. cotiara*. In SVSPs, eight toxin isoforms were more highly expressed in *B. fonsecai*. Interestingly, the SVSP from *B. cotiara* previously described as cotirianase [30] was detected in our analysis. An orthologous toxin with a similar expression level was also detected in *B. fonsecai*. Moreover, we detected four CTL isoforms with higher expression in *B. fonsecai*, whereas one BPP and the NGF presented a higher expression in *B. cotiara*. Together these findings proved evidence of transcriptomic variation, which may be related to the activity of regulatory elements, such as transcription factors.

### 2.2. Proteome Analysis

We generated venom proteomic data and used the toxin sequences identified in the venom gland transcriptome assemblies as a database for protein identification. Comparisons among the resulting proteomes revealed discrepancies in the abundances of many toxins quantified in the transcriptome (Figure 3). For both species, BPPs were not detected in the proteomic data since its precursor is naturally processed into peptides [31] and these toxins are only detected through peptidomic analysis. In *B. cotiara*, most of the proteins encoded by toxin transcripts were detected proteomically (i.e., 49 from 54 toxins) with only five toxins not identified in the proteome (i.e., two CTLs, two SVSPs, and one SVMPII; Table S4 in Supplementary Materials). The unidentified toxins were lowly expressed in the transcriptome, which may be related to the absence in the proteomic data (Tables S3 and S4 in Supplementary Materials). For the detected toxins, we observed that the proteome presented an increased abundance of SVMPs, SVSPs, and LAO and a decreased abundance of PLA2, CTLs, and VEGF when compared to the transcriptome data. The SVMPs, CTLs, and SVSPs were the major components of venom proteome and accounted for 51%, 22.4%, and 14.3%, respectively. As minor components, we observed that LAO, CRISP, VEGF-F, 5NUC, and PLB represented 4.0%, 2.8%, 1.9%, 1.3%, and 1.1%, respectively. Interestingly, PLA2 represented around 1% of the *B. cotiara* proteome, which indicates lower representativeness in the venom composition of *B. cotiara*. Despite the low amount of PLA2 detected in our proteomic data, two previous reports revealed that intraspecific variation may also be detected in the venom composition of *B. cotiara*, whereas one report detected total absence of PLA2 in the venom of a specimen from an unknown location of Brazil and other report detected high abundance of PLA2 in a specimen from Misiones, Argentina [14]. Further studies that focus on expanding the regional diversity of individuals analyzed may provide better insight on the intraspecific diversity of venom composition in *B. cotiara*. Unfortunately, we were not able to perform intraspecific variation analysis within *B. cotiara* proteomic data once we had an insufficient amount of venom from the individual SB0266 to use in proteomic analysis.

The venom proteome of *B. fonsecai* presented the majority of proteins encoded by toxin transcripts (i.e., 44 from 47 toxins), except for three toxins that are lowly expressed in the transcriptome (i.e., HYAL, NGF, and one SVMPII; Tables S3 and S4 in Supplementary Materials). Despite these unidentified toxins, the proteome presented an increased abundance of SVMPs, SVSPs, LAO, and CRISP and a decreased abundance of PLA2 and CTLs (Figure 3) in comparison to the transcriptome. We noticed that SVMPs, CTLs, and SVSPs were the major components accounting for 44.9%, 17.4%, and 15.1%, respectively. As minor components, we observed that CRISP, LAO, venom phosphodiesterase (DIESTER), PLB, PLA2, and VEGF-F represented 4.3%, 4.1%, 3.6%, 3.0%, 2.95%, and 2.27%, respectively. The proteome revealed that the *B. fonsecai* proteome presented a lower level of intraspecific variation similar to that observed in the transcriptome (Figure S3 in Supplementary Materials).

The interspecific variation of proteomes revealed that some SVMPs, SVSPs, CTLs, and PLA2 presented differences in abundances, whereas all other toxins presented conserved expression (Figure 3; Table S4 in Supplementary Materials). Similar to the differentially expressed toxins in the transcriptome, we observed that the proteome shows different abundances of PLA2, SVMPs, SVSPs, and CTLs between species. Looking at the toxin family abundance, *B. fonsecai* presented a higher abundance of PLA2, PLB, and SVMPIII, whereas *B. cotiara* presented a higher abundance of SVMPII and CTL. On the other hand, most of the differentially expressed toxin isoforms tend to be more abundant in the venom of *B. fonsecai* than in *B. cotiara*, indicating that this venom may present a higher complexity.

The comparative analysis of the venom composition revealed that the primary axis of variation between these species relies on PLA2. We found that *B. fonsecai* presented a higher abundance of PLA2 than *B. cotiara*, which corroborates previous findings [13]. We also showed that PLA2 is not totally absent in *B. cotiara*, which is in accordance with a recent report [14]. Moreover, we noticed a higher expression of 8 isoforms of each SVMP and SVSP, 4 isoforms of CTL in *B. fonsecai*, and a higher expression of BPP and the NGF in *B. cotiara*. These differences may be a result of the evolution of toxins within modules of expression, which is a feature that allows rapid transitions between phenotypes while avoiding or minimizing the occurrence of low-fitness intermediates [32].

**Figure 4:**
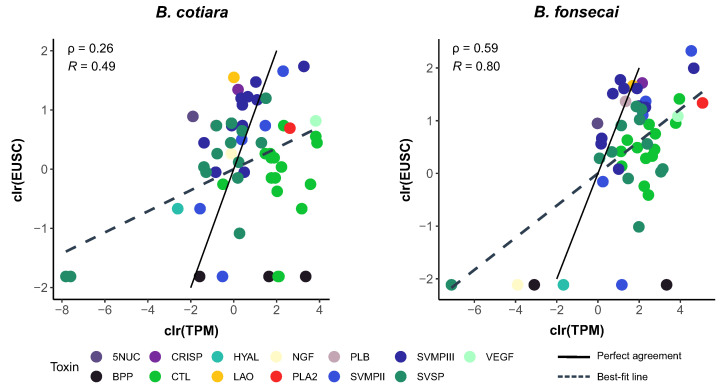
Comparison between levels of transcripts (TPM) and protein abundance (normalized EUSC-Exclusive Unique Spectrum Count) of *Bothrops cotiara* and *B. fonsecai*. All data were centered log-ratio transformed (clr). Black lines indicate a perfect agreement between transcript and protein abundances. Dashed lines indicate the best-fit line between transcript and protein abundances. ρ—Spearman’s rank correlation coefficient. *R*—Pearson’s correlation coefficient. TPM—Transcripts Per Million. EUSC—Exclusive Unique Spectra Counts.

Interestingly, we noticed that the protein quantity of some isoforms was correlated with the amount of mRNA in the transcriptome; however, some isoforms presented discrepancies indicating putative roles for regulatory mechanisms shaping the translation efficiency (Figure 4; Figure S4 in Supplementary Materials). The proteome data showed a lower quantity of CTLs than in the transcriptome for both species, whereas the amount of SVMPs was higher in the proteome than in the transcriptome. We also detected that most of the toxin orthologs presented a slightly higher abundance in *B. fonsecai* than in *B. cotiara* (Figure 3). Comparing the transcriptomic and proteomic abundances within each species revealed that *B. fonsecai* has a strong positive correlation (ρ = 0.59; *R* = 0.80; Figure 4), whereas *B. cotiara* presents a weak positive correlation (ρ = 0.26; *R* = 0.49; Figure 4). The strong positive correlation observed in *B. fonsecai* is similar to that observed in other snakes [18,33], which are higher than that obtained in *B. cotiara*. Considering that the protein identification was performed using the species consensus transcriptome rather than individual transcriptomes, some discrepancies in correlation may be expected. However, the weaker correlation observed in *B. cotiara*, when compared to other snakes (0.47 ≤ ρ ≤ 0.89; 0.53 ≤ *R* ≤ 0.92), suggests that posttranscriptional regulatory mechanisms may be shaping the amount of final toxin proteins produced by *B. cotiara*.

### 2.3. Functional Differences in the Toxic Activities of *B. cotiara* and *B. fonsecai* Venoms

**Figure 5:**
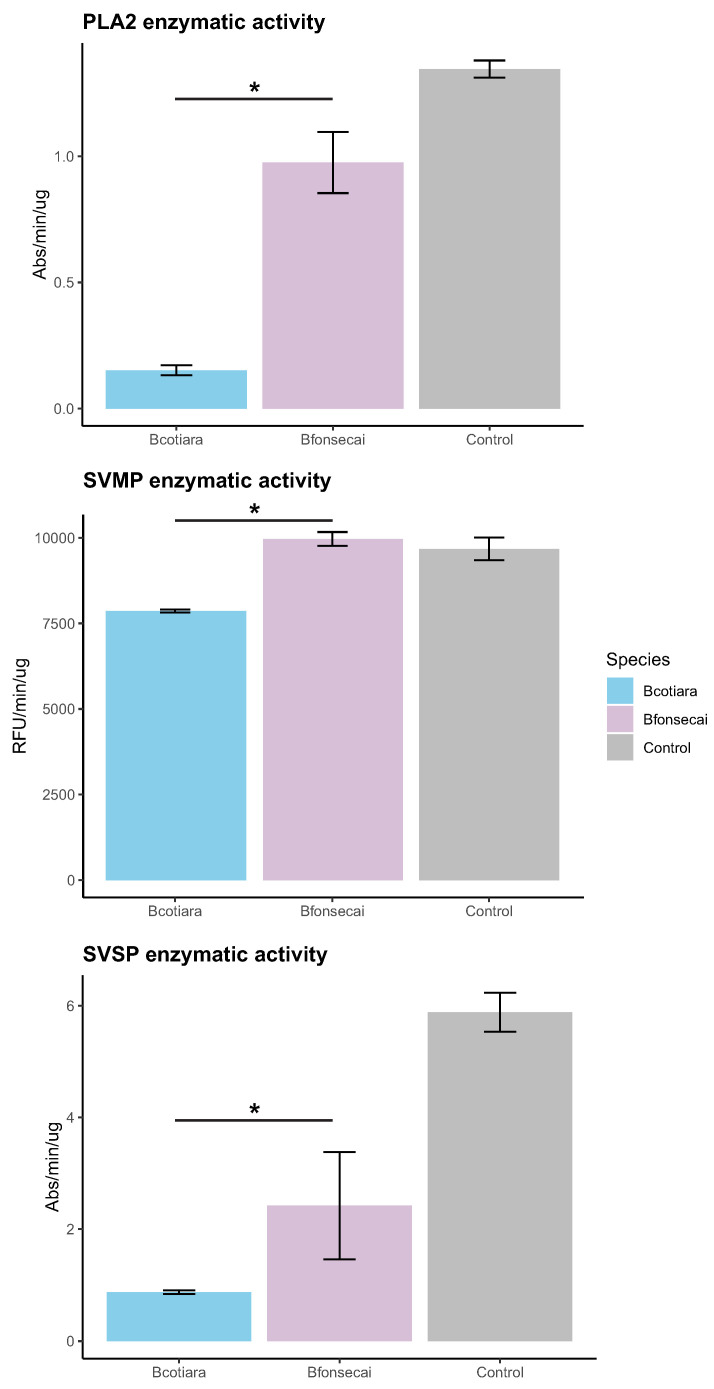
PLA2, SVMP, and SVSP enzymatic assays of *Bothrops cotiara* and *B. fonsecai* venoms. The asterisks indicate statistically significant differences (*p*-value < 0.05).

Considering that the major source of variability in venom composition was related to the abundance of PLA2 and a slight difference in SVMPs and SVSPs, we tested the enzymatic activities of PLA2, SVMP, and SVSP (Figure 5). The enzymatic activity of PLA2 was significantly lower in *B. cotiara* when compared to *B. fonsecai* (*p*-value = 2.92 × 10${}^{-6}$), as previously reported [15]. The enzymatic activity of SVMPs and SVSPs was slightly higher in *B. fonsecai* (*p*-values = 5.25 × 10${}^{-5}$ and 1.25 × 10${}^{-4}$, respectively). The higher activity of SVMPs in *B. fonsecai* may be related to the higher abundance of most SVMP-III isoforms present in its venom, whereas the higher activity of SVSPs in *B. fonsecai* may be related to the higher abundance of some isoforms of SVSPs in its proteome (Figure 3).

In this context, the results from the enzymatic assays agree with those obtained in the proteomic analysis, which corroborates previous comparative findings related to these toxin activities in both species [15]. Despite the observed differences in toxin activities, the venoms of *B. cotiara* and *B. fonsecai* were shown to present similar toxicity [15]. Another report also showed that the biological effects of *B. fonsecai* venom may be related to the abundance of certain SVMPs and SVSPs rather than PLA2 [34], which may be the same case in *B. cotiara*. In fact, it was well discussed that the amount of PLA2 has little effect on the lethality and toxicity of snake venoms when injected in rodents (reviewed in [35]) and that the presence of PLA2 may be correlated to a generalist diet [35]. In this context, we hypothesize that the expression of PLA2 may be experiencing neutral or nearly neutral evolutionary processes, such as drift, while the expression of other venom components, such as SVMPs and SVSPs, may be experiencing a stabilizing selection. These evolutionary processes may be acting to shape the venom composition observed with no dramatic changes in the venom function of both species. However, further functional assays focusing on comparing the biological effects of the venom and its specific components from both species using *in vivo* rodents as models must be performed to evaluate such hypotheses.

### 2.4. Evaluating Regulatory Mechanisms in the Venom Gland

**Figure 6:**
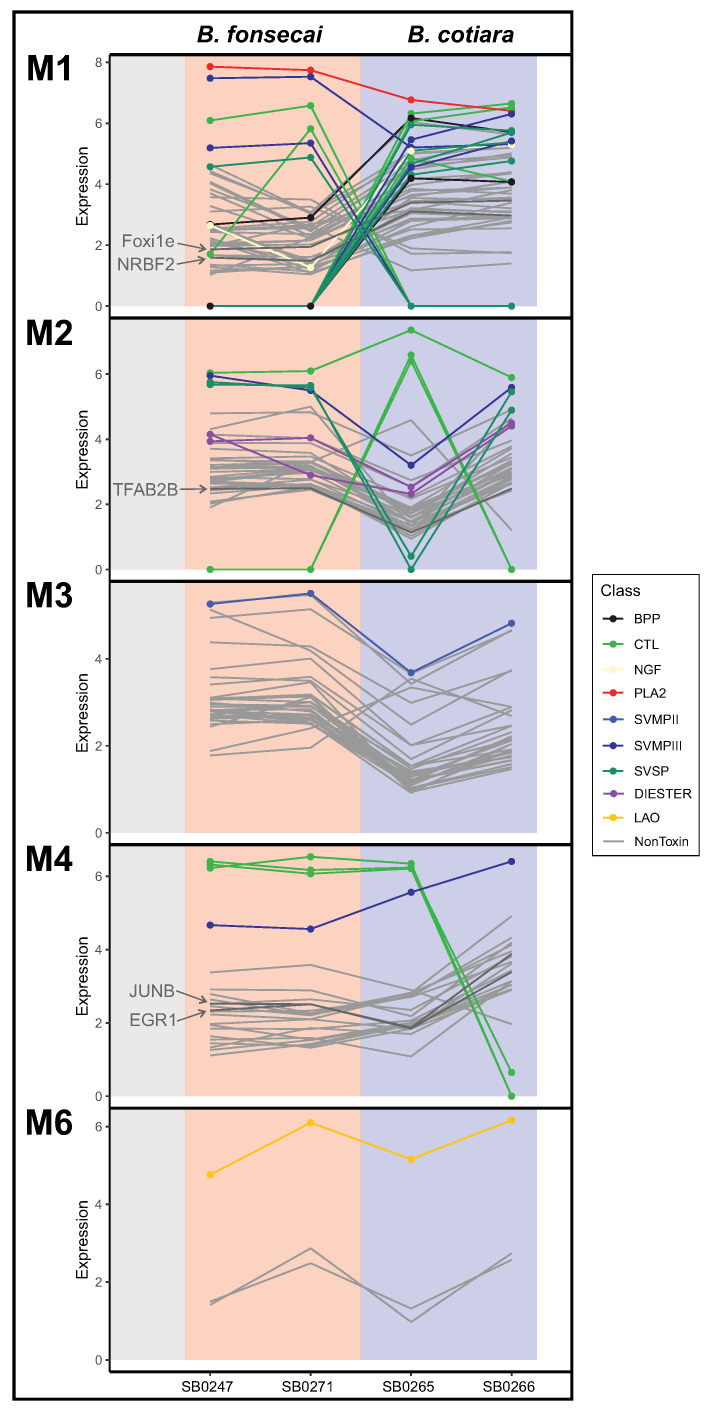
Expression profiles for five coexpression modules containing toxin transcripts from the seven modules identified by CEMiTool. Each line represents a transcript and its change in expression across samples. Toxins assigned to each module are colored by class. Nontoxins associated with a module are shown as light gray lines and transcription factors are shown as dark gray lines and labelled.

Taking into consideration the interspecific variation observed between *B. cotiara* and *B. fonsecai*, we performed a modularity analysis to detect modules of coexpression to capture the transcriptional regulatory mechanisms that may be modulating the venom phenotype observed and if the modular toxin expression may explain the variations detected. The detection of coexpression modules allows to identify correlations that indicate which genes are active in the same biological processes and pathways that led to the final phenotype; however, when there is no genomic and epigenetics data available, it does not confer information about causality or allow to distinguish between regulatory and regulated genes. Here, we performed the detection of coexpression modules to test if the modular expression is a driver of the venom variation observed between *B. cotiara* and *B. fonsecai* and to infer candidate regulatory genes that may be shaping the venom phenotype observed in both species. After transcript filtering performed by CEMiTool, we detected 154 transcripts grouped into 7 coexpression modules. Of these, 33 were toxin transcripts and 121 were nontoxin transcripts (Table S5 in Supplementary Materials). Toxin transcripts were present in 5 of the 7 modules detected (Figure 6). From the nontoxin transcripts, we were able to detect 5 transcription factors (TFs) that may be associated with the transcriptional regulation of toxins and/or nontoxins within each module (Figure 6). Most of the toxins presenting differential expression between species segregated into module 1, where we detected the TFs forkhead box protein I1-ema (Foxi1e) and nuclear receptor-binding factor 2 (NRBF2). These TFs are known to be transcriptional activators, which indicates that they may be activating certain toxin transcripts when they have its expression upregulated. In fact, enriched binding site motifs for members of the forkhead proteins were detected in the promoter/TSS region of SVMPs genes in the tiger rattlesnake and it was detected as highly expressed in the venom gland of the prairie rattlesnake [36,37]. We also detected the transcription factor AP-2 beta (TFAB2B) in this module, indicating that this TF may be shaping the toxin transcript abundance of this module as well. In module 4, we detected the TFs transcription factor jun-B (JUNB) and early growth response protein 1 (EGR1) that may be acting to regulate the expression of the CTLs and/or the SVMPIII grouped together within this module. Enriched binding sites of JUNB were also detected in the promoter/TSS regions of toxin genes in the tiger rattlesnake [37]. Our analysis revealed that toxin transcripts with differential expression between species may segregate within modules and allow the detection of TFs that may be shaping its transcription levels. However, further experiments using the genome sequence of these species may help to detect the binding sites of such TFs in the promoter regions of toxins to help understand its regulatory roles on toxin transcription.

**Figure 7:**
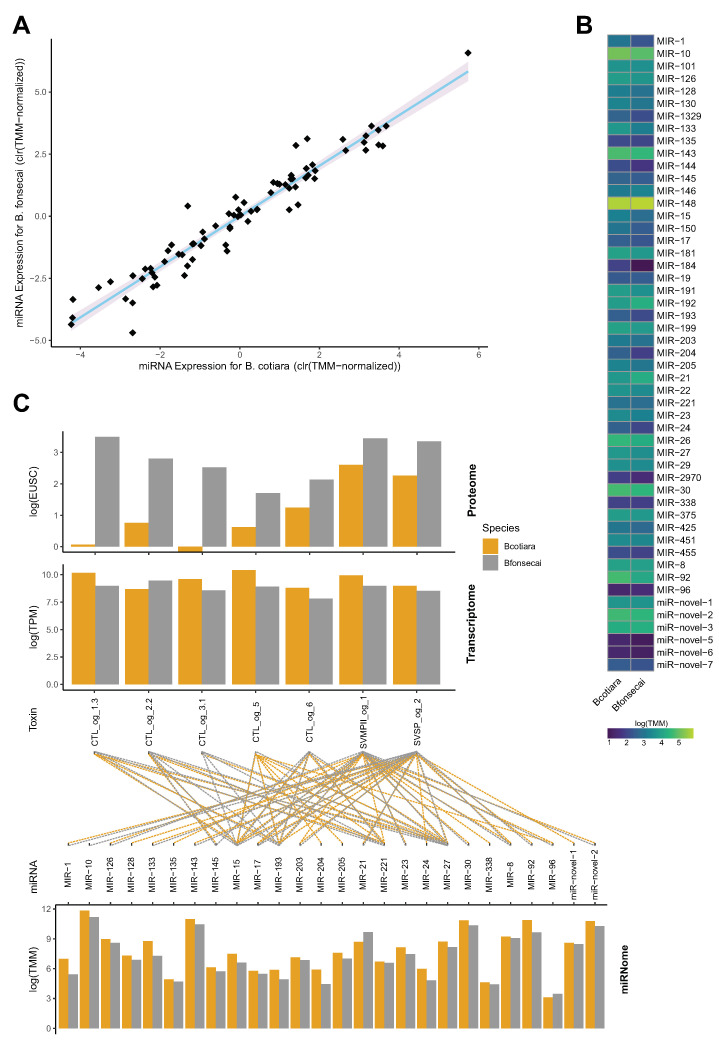
Venom gland miRNome characterization and its putative toxin targets. (**A**) Chart showing the interspecific variation profile of miRNA expression between both species. (**B**) Chart showing the miRNAs expression profile in both species. (**C**) Charts showing the toxin transcripts that may be under the action of miRNAs with expression levels in transcriptome and proteome at the top and middle. The miRNAs that may be modulating those toxin genes and their expression levels are shown at the bottom; the dashed lines represent the interaction between miRNA and toxin transcript. TMM—Trimmed Mean of M-values. TPM—Transcripts Per Million. EUSC—Exclusive Unique Spectra Counts.

Although posttranscriptional regulatory mechanisms often do not appear to contribute significantly to the final venom phenotype [33], some studies have shown that miRNAs may mediate the ontogenetic shift of specific toxin isoforms in *Crotalus* [23,24] and sea snake species [25]. To test if post-transcriptional regulatory elements shape the translation efficiency of toxin transcripts in *B. cotiara* and *B. fonsecai*, we characterize the whole set of miRNAs expressed in the venom glands of both species. We were able to detect 72 miRNA transcripts with known orthologous miRNA genes and 6 putative novel miRNAs representing 45 miRNA families (Figure 7; Table S6 in Supplementary Materials). Most of the miRNAs detected presented a similar expression profile between species, whereas some miRNAs presented a likely differential expression (Figure 7A,B). Target prediction analysis revealed that miRNAs in both species are likely to bind to similar 3’UTR regions of the same toxin transcripts due to the similarity among sequences. However, miRNAs with slightly higher expression in *B. cotiara* presented potential binding sites in the 3’UTR of toxin transcripts with low translation efficiency (Figure 7C; Table S7 in Supplementary Materials), which indicates a putative role for these miRNAs in *B. cotiara*. In this sense, our data suggest that miRNAs are not strongly affecting the differences observed between both species, but some miRNAs may be acting together to fine-tune the final protein abundance observed of some toxins. Then, miRNAs may represent regulatory elements of venom GRNs adjusting the final venom phenotype, but further functional experiments are necessary to confirm such interactions and better clarify the functional roles of miRNAs in toxin production.

In summary, our analysis revealed that most of the differences observed between *B. cotiara* and *B. fonsecai* may be explained by variation through modular toxin expression in venom glands [19]. Module M1 contains most of the differentially expressed toxins between the species, such as PLA2, SVMPs, SVSPs, and CTLs, suggesting a common axis of regulation underlying interspecific venom differentiation. Modules M2 and M4 have type III SVMPs and CTLs and indicate the isoforms within each module are under a concerted evolution process to shape the final venom phenotype observed. Module M3 contains only SVMPII-5 and other nontoxin genes, which may indicate relaxed expression evolution of this toxin. The detected modules of coexpression allow us to hypothesize that the expression of toxin and nontoxin genes within each module are under a similar evolutionary pressure that may be shaping the final venom phenotype observed in *B. cotiara* and *B. fonsecai*. The modularity analysis also revealed some transcription factors associated with specific submodules that may be regulating the toxin transcription. This indicates that modularity approaches may be useful to detect potential regulators of toxin transcription in venom gland transcriptome analysis.

### 2.5. Toxin Gene Family Analysis

**Figure 8:**
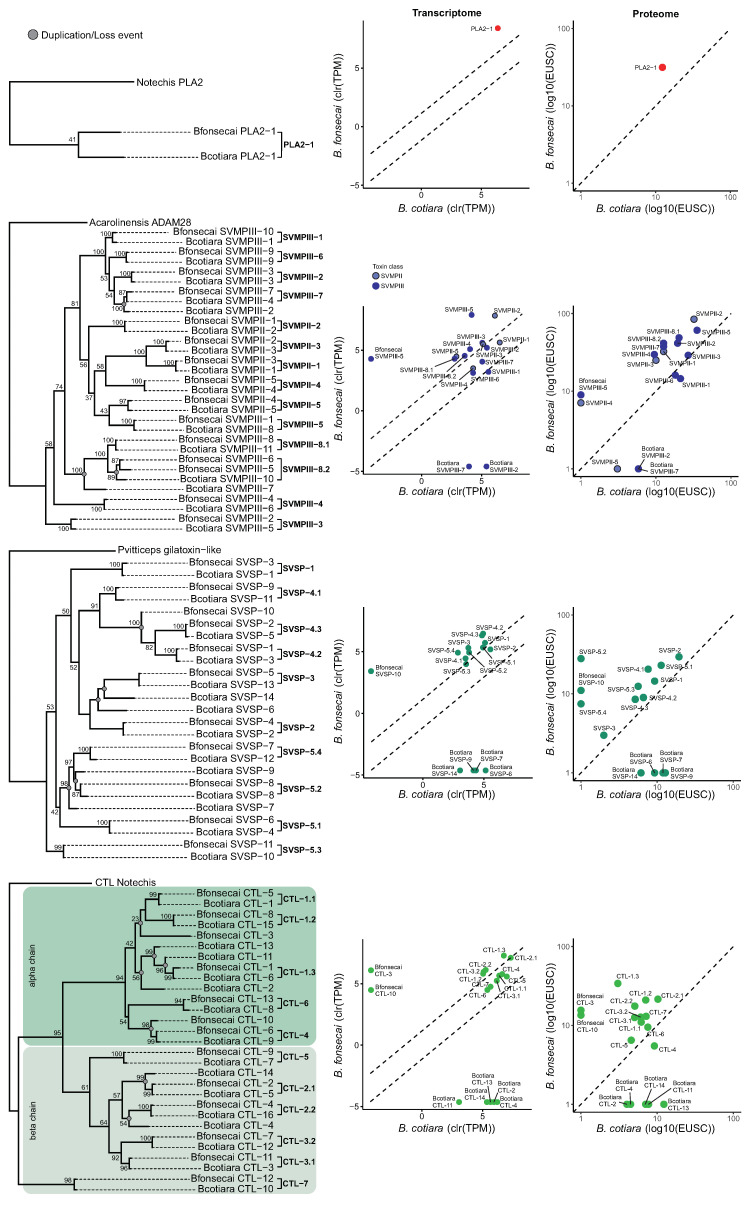
Toxin family phylogenies and interspecific variation of transcriptome and proteome of PLA2, SVMPs, SVSPs, and CTLs. The one-to-one orthology is marked by brackets in the phylogeny and putative duplication/loss events are marked by gray circles in the phylogeny. The transcriptome expression plots are based on average expression of each toxin for each species and dashed lines denote the 99% confidence interval established by nontoxin expression. The dashed line in the proteome expression plots indicates a hypothetical correspondence of protein abundances between species. TPM—Transcripts Per Million. EUSC—Exclusive Unique Spectra Counts.

We inferred phylogenies for four highly expressed and diverse toxin families to better understand the dynamics of duplications and losses acting to shape the venom phenotype (Figure 8). In the four toxin families analyzed, we identified one-to-one orthologs for the majority of toxins, although expression levels were not necessarily conserved. However, SVMP, SVSP, and CTL families presented putative duplication/loss events.

In the PLA2, we detected one transcript for each species, which were identified as one-to-one orthologs and showed higher expression in *B. fonsecai*. The PLA2 identified in both species is the acidic D49 PLA2 type. We noticed a higher expression of PLA2 in transcriptome and proteome data of *B. fonsecai*. This PLA2 was identified in module M1 of the modularity analysis, which also contains two TFs that may be acting on the expression level of this toxin.

In SVMPs, we identified five SVMPII transcripts in each species that were identified as one-to-one orthologs and nine SVMPIII transcripts in each species that were identified as one-to-one orthologs. We also identified duplication/loss events in SVMPIII in both species. In SVMPIIs, two SVMPII transcripts showed a distinct protein expression profile; specifically, SVMPII-4 was detected in the proteome of *B. fonsecai* and SVMPII-5 was specifically detected in the proteome of *B. cotiara*. Moreover, SVMPII-1, which showed similar expression between both species in the transcriptome data, presented higher abundance in the proteome of *B. fonsecai*, which indicates that this turnover may be related to the miRNAs binding sites predicted in its 3’UTR that presents higher expression in *B. cotiara*. In SVMPIIIs, all one-to-one orthologs were expressed in both species. We noticed that SVMPIII-1 presented higher expression in *B. cotiara* and was identified in module M4 of the modularity analysis. On the other hand, SVMPIII-4 and SVMPIII-5 presented higher expression in *B. fonsecai* and were detected in modules M2 and M1 of the modularity analysis, respectively. Interestingly, we detected that SVMPII-5 may represent a fusion of sequences from SVMPII-2 and SVMPIII-5 in both species. Considering that a fusion of genomic sequences from SVMPII and SVMPIII resulted in an SVMPII locus in the *Crotalus atrox* [38], we hypothesize that a similar event took place in a common ancestor of these *Bothrops* species to generate the SVMPII-5 transcript detected.

In the SVSPs, we detected ten transcripts with one-to-one orthology and three putative duplication/loss events. We noticed a tendency of higher expression in the transcriptome and proteome *B. fonsecai* for most of the SVSPs. SVSP-5.2 and SVSP-5.4 were detected in module M2 of the modularity analysis. The SVSP-2, which is highly similar to the previously reported cotiarinase [30], presents similar expression in the transcriptome and higher expression in the proteome of *B. fonsecai*, which may reflect the modulation of miRNAs that presents higher expression in *B. cotiara* and may be regulating the protein output of this isoform.

In the CTLs, we inferred 12 one-to-one orthology and seven putative duplication/loss events. In general, most of the one-to-one orthologs presented similar expression profiles in the transcriptome but slightly higher abundance in the proteome of *B. fonsecai*. CTL-1.2, CTL-2.2, CTL-3.2, and CTL-5 were detected in modules M2 and M4 in the modularity analysis. On the other hand, CTL-1.3, CTL-2.2, CTL-3.1, CTL-5, and CTL-6 may be affected by the activity of miRNAs regulating the final output observed for these isoforms in *B. cotiara*. Moreover, the CTL gene tree revealed that the CTLs detected in each species were grouped into alpha and beta chains (Figure 8; Figure S5 in Supplementary Materials). We detected that the homologs of alpha and beta chains of Bothrojaracin, a potent thrombin inhibitor isolated from *B. jararaca* venom [39], are similarly expressed in both species (i.e., CTL-1.1 and CTL-3.1, respectively).

We detected several putative duplication or loss events in toxin genes, but further genomic studies involving both species are necessary to confirm such events and give a better picture of the evolutionary paths of toxin genes. However, duplication and losses were reportedly described in toxin genomic regions inter- and intraspecifically in snake species [19,37,38], which suggests that the events detected in our analysis may reveal bonafide duplication/loss events.

### 2.6. Toxin Sequence Diversification Analyses

**Figure 9:**
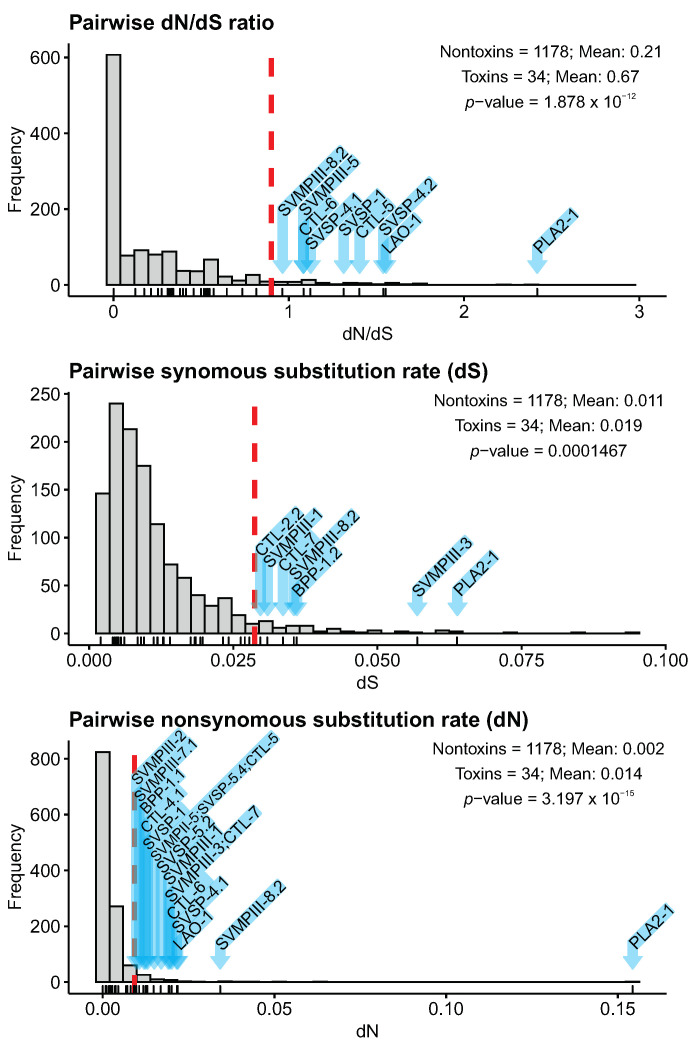
Distribution of pairwise dN/dS ratios, synonymous substitution rates, and nonsynonymous substitution rates of toxins and nontoxins orthologous coding sequences (CDSs). Dashed red lines denote the 95th percentiles based on distribution. Lines beneath plots indicate toxins, and toxins with values greater than the 95th percentile are marked with blue arrows.

We compared the pairwise synonymous substitution rates (dS), nonsynonymous substitution rates (dN), and dN/dS ratios (ω) between the toxin and nontoxin orthologs to better understand the selective regimes of toxin genes present in the venom of both species. Toxins had a higher ω ratio than nontoxins with mean ω values around 0.67 and eight toxins presented ω values greater than 1, indicating positive selection (Figure 9). We also detected that synonymous and nonsynonymous substitution rates were significantly higher in toxins when compared to nontoxins. Moreover, seven toxins were found to have synonymous substitution above the 95th percentile, whereas 17 toxins presented nonsynonymous substitution above the 95th percentile. Interestingly, the PLA2 presented the highest ω value (ω = 2.41), as well as the highest synonymous and nonsynonymous values among toxins.

In general, we noticed that ω values of most toxins were below one (i.e., 26 from 34 toxins), which indicates that these toxins may be evolving under a model of relaxed purifying selection between *B. cotiara* and *B. fonsecai*. The eight toxins presenting a ω value greater than 1 are under a positive selective pressure and belong to 4 toxin families with high representativeness in the venom composition of *B. cotiara* and *B. fonsecai* (i.e., SVMP, SVSP, CTL, and LAO) and to one family differentially represented in the venom of both species (i.e., PLA2). Our results are in accordance with previous interspecific comparisons which showed that most toxins present a ω higher than the average of nontoxins but only some toxins presented a ω greater than 1 [16,19].

### 2.7. Are the PLA2 Genes of *B. cotiara* and *B. fonsecai* Direct Orthologs?

**Figure 10:**
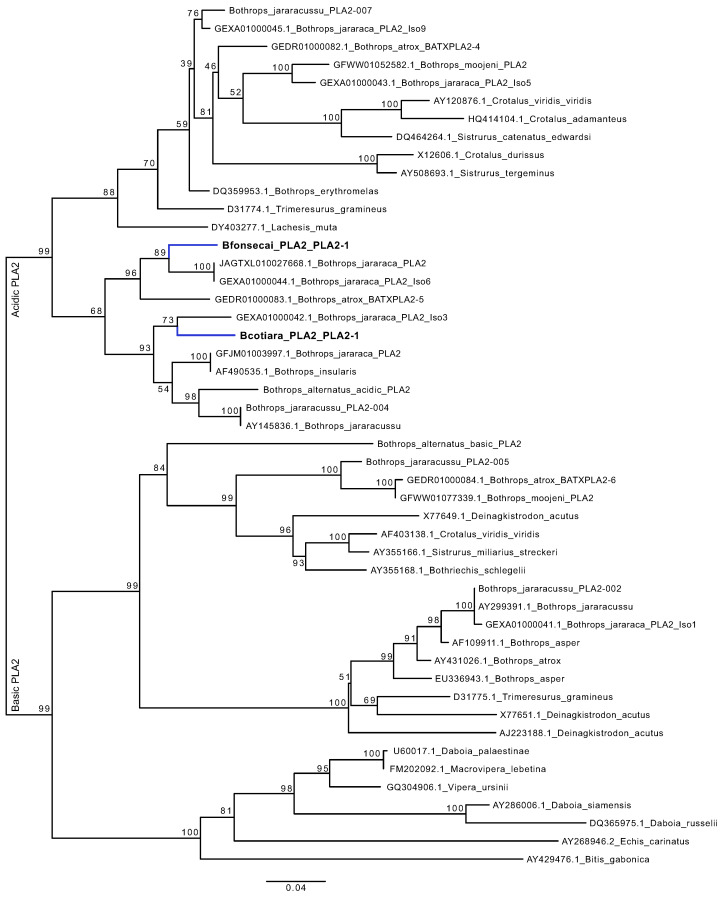
Phylogeny of the coding sequence of PLA2 genes of several *Bothrops* and Viper species. The PLA2 genes from *B. cotiara* and *B. fonsecai* are shown in bold and branches are highlighted blue. The support values of bootstrap are shown at tree nodes.

Considering the high mutation rate observed in the interspecific PLA2 gene comparison, we performed an in-depth analysis to better understand the evolution of this toxin gene in both species. Specifically, we performed a phylogenetic analysis using the coding sequences (CDSs) of PLA2 available from other *Bothrops* and viper species (Figure 10). This analysis revealed that the PLA2s identified in both species, which were characterized as orthologous by OrthoFinder and present a high mutation rate, may represent a preexisting paralogous relationship (alloparalogs) rather than an orthology relationship. However, we are not able to confirm either relationship based only on the CDS and/or protein sequences. In this sense, we hypothesize two evolutionary scenarios that may explain the PLA2 evolution in the species analyzed (Figure S6 in Supplementary Materials). In both scenarios, the most recent common ancestor of *Bothrops* may have possessed at least six copies of PLA2 genes (i.e., two basic and four acidic) and successive lineage-specific losses took place during *Bothrops* evolution and speciation events. In the first scenario, the PLA2 genes in *B. cotiara* and *B. fonsecai* are true orthologous where the same locus was maintained in both species and experiences a high mutation rate and strong positive selection. This high mutation rate led to the PLA2 observed in *B. fonsecai* to converge with similar PLA2 sequences identified in other *Bothrops* species. In the second scenario, the PLA2 gene detected in *B. cotiara* and *B. fonsecai* species may be in distinct loci, corresponding to a paralogous relationship. This would explain the nesting of these genes in different branches and the high mutation rate observed among them. Moreover, these results indicate high variation in the PLA2 genomic region of *Bothrops* species. In fact, other transcriptomic studies using *Bothrops* as the model species revealed a distinct number of PLA2 genes, which indicates interspecific variation in the presence/absence of toxin genes in the genomes of *Bothrops* species. Moreover, intraspecific variation in PLA2 loci was observed in *B. jararaca*, whereas one study using transcriptomic data reported five copies of PLA2 genes in one individual [26] and another study using genomic data reported one copy of PLA2 gene in another individual [40]. Interestingly, this issue was previously observed in rattlesnakes where successive losses of PLA2 genes may have occurred during the evolution of *Crotalus* species inter- and intraspecifically [37,41]. In this sense, we believe that the second hypothesis may represent a bonafide explanation for the results obtained in the present study, but further experiments, such as sequencing the genomic loci of PLA2 genes in both species, must be performed to confirm such a hypothesis.

This question highlights the necessity of critical evaluation of orthology identification when studying toxin genes as results obtained under assumptions typical of most genes may lead to erroneous conclusions. Moreover, it also underscores the fact that deciphering the genomic context of the toxin genes being studied may improve the understanding of the evolution of toxin genes. In this sense, the results obtained in the present study suggest that sequencing the genome of both species will help clarify the evolution of PLA2s in *Bothrops* species.

## 3. Conclusions

In the present study, we demonstrated that the primary difference in the venom composition of *B. cotiara* and *B. fonsecai* is related to the expression of PLA2; however, the venoms of both species present similar toxicity and lethality, which may be the result of the similar expression level of specific SVMPs and SVSPs isoforms. We showed that *B. cotiara* possesses and expresses a PLA2 gene in accordance with recent reports. However, the PLA2s detected in *B. cotiara* and *B. fonsecai* are likely different paralogs rather than direct orthologs, though further genomic analysis are necessary to confirm these inferences and improve our understanding of toxin evolution in these species. Our analysis also revealed that most of the differences observed in toxin expression between *B. cotiara* and *B. fonsecai* may be explained by modular toxin expression through the regulation of transcription by specific transcription factors. This suggests that the detection of coexpression modules may be useful to identify potential regulators of toxin expression in venom glands. We also showed that post-transcriptional mechanisms, such as miRNAs, likely do not cause major changes in venom composition but may play a role in fine-tuning the final protein output of specific toxin isoforms.

## 4. Materials and Methods

### 4.1. Sample Collection

Two adult specimens of *B. cotiara* were collected in January 2018 in Santa Catarina state, Brazil. Both individuals were sampled near Urubici city. Two adult specimens of *B. fonsecai* were collected in January 2018 in São Paulo state, Brazil, with one individual sampled near Campos do Jordão city and the other sampled near São Bento do Sapucaí city. For all snakes, venom was collected by allowing the snake to bite a sterile cup, and venom glands were excised for transcriptomics after four days when transcription is maximized [42]. The specimens were euthanized with single-step sodium pentobarbital (100 mg/kg) injection following standard approved AVMA guidelines. Venom glands were transferred to RNAlater (Ambion, Austin, TX, USA) and stored at −80 ${}^{\circ }$C. The snakes were handled and collected in accordance with the guidelines of the Brazilian College for Animal Experimentation (COBEA) and under Protocol Number 4479020217 from the Ethics Committee on Animal Use of the Butantan Institute (CEUAIB). The specimens used in the present study were registered with the Number A6E9C41 in the Brazilian National System for the Management of Genetic Heritage and Associated Traditional Knowledge (SisGen).

### 4.2. RNA Extraction and Sequencing Protocol

Total RNA from the venom gland was extracted using Trizol Reagent (Invitrogen, Waltham, MA, USA), following the manufacturer’s protocol. The RNA concentration and contamination level were measured by UV absorbance using NanoDrop 1000 (Thermo Scientific, Waltham, MA, USA) and RNA integrity was assessed with the Agilent 2100 Bioanalyzer (Agilent Technologies, Santa Clara, CA, USA).

Next, mRNA was purified from the total RNA using the Dynabeads^®^ mRNA DIRECT kit (Ambion) and was used to prepare independent cDNA libraries for each venom gland from each snake. The cDNA libraries were prepared following the protocol for TruSeq™ RNA Sample Preparation Kits v2 (Illumina, San Diego, CA, USA) and sequenced using the HiSeq1500 platform (Illumina), generating strand-specific paired-end reads (2 × 150 bp).

We also used the total RNA to sequence miRNAs expressed in the venom gland. The small RNA library was generated using the Illumina Truseq™ Small RNA Preparation kit, following Illumina’s TruSeq™ Small RNA Sample Preparation Guide (Illumina). The purified cDNA library was used for cluster generation on Illumina’s Cluster Station and then sequenced on the HiSeq1500 platform (Illumina).

### 4.3. Transcriptome Assembly, Annotation, and Quantification

Adapters and low-quality reads were trimmed using Trim Galore! (v0.4.4; https://github.com/FelixKrueger/TrimGalore, accessed on 25 January 2022) with parameters set to remove reads with phred scores less than five and a length less than 75 bp. The paired-end reads were merged using PEAR [43] and then de novo assembled following recommendations to use several tools and parameters to improve assembly completeness [44,45]. Specifically, we used five assemblers: Trinity (kmer parameter set to 31; [46]), Extender (overlap parameter set to 120 and 150; [47]), NGen (using the default parameters; Lasergene DNAStar, Madison, WI, USA), Bridger (kmer parameter set to 30; [48]), and rnaSPAdes (kmer parameter set to 31, 75, and 127; [49]). All assemblies were combined and clustered with a 100% identity using the “RemoveRedundancy.py” script available in the repository of ToxCodAn (https://github.com/pedronachtigall/ToxCodAn, accessed on 25 January 2022) to remove redundancy.

The annotation of toxins was performed using ToxCodAn (v1.0; [28]). We combined the resulting toxin (redundancy-filtered) and putative toxin (SP-filtered) CDSs into a single file. Next, we performed a manual curation and eliminated chimeric transcripts using the custom ChimeraKiller script (v0.7.4; https://github.com/masonaj157/ChimeraKiller, accessed on 25 January 2022). Removing chimeric sequences further reduces the number of annotated transcripts (false positives) and removes putatively spurious transcripts from the final transcriptome. Finally, we clustered the cleaned toxin CDSs with 99% similarity using cd-hit [50] to reduce redundancy of repeated transcripts and group allelic variation at a single locus.

The annotation of nontoxins was performed using the nontoxin transcripts output by ToxCodAn with its CDSs predicted using CodAn (v1.0; [51]) with the full model designed for vertebrates. Predicted CDSs were then BLAST searched against two protein databases: (1) a specific protein database from ToxCodAn (proteinDB; https://github.com/pedronachtigall/ToxCodAn, accessed on 25 January 2022); and (2) the Swissprot database (ftp://ftp.ncbi.nlm.nih.gov/blast/db/swissprot.tar.gz, accessed on 25 January 2022). We then performed an HMM search of the CDSs not annotated in the BLAST search step using hmmsearch (HMMER v3.2.1; http://hmmer.org/, accessed on 25 January 2022) with the vertebrate Hidden Markov Models (HMM) from BUSCO ([52]) and hmmscan (HMMER v3.2.1; http://hmmer.org/, accessed on 25 January 2022) with the HMM models from Pfam (https://pfam.xfam.org/, accessed on 25 January 2022).

The final nontoxins were combined with the final ToxCodAn-annotated toxins and clustered with 99% similarity using cd-hit. To create a species consensus transcriptome, we combined the individual’s transcriptomes of each species together and clustered transcripts at 98% similarity.

### 4.4. Expression Analyses and Ortholog Identification

We performed transcript quantification by mapping the reads against the species consensus transcriptome using Bowtie2 (v2.3.5; [53]) and calculating the relative expression with RSEM (v1.3.1; [54]). We assessed the intraspecific variation in expression using the species-specific data sets for *B. cotiara* and *B. fonsecai*. Due to a limited intraspecific sampling, we generated a pairwise null distribution of expression divergence based on nontoxin expression to identify putative outliers that may represent divergence in expression level [55]. The data was centered log-ratio (clr) transformed to normalize the expression distributions while accounting for the compositional nature of relative expression values (i.e., TPM). The toxin transcripts with pairwise divergence in expression outside the 99th percentile of the centered log-ratio transformed distribution of nontoxins were considered outliers that may represent differentially expressed toxins.

To perform interspecific variation analysis, we first inferred orthology among toxin and nontoxin transcripts of both species using OrthoFinder (v2.3.14; [56]). OrthoFinder allows the identification of groups of sequences derived from a single gene that share the same common ancestor of compared species (i.e., orthogroups), and also identifies conserved orthologs within orthogroups. In this sense, OrthoFinder output permits the identification of orthology among the analyzed sequences, which allows a comparative analysis between species.

For nontoxins, we filtered low expressed transcripts by keeping only genes with TPM higher than 1 in both individuals of each species (i.e., genes with TPM lower or equal to 1 in one individual were removed from downstream analysis). Then, we applied OrthoFinder to detect orthogroups among nontoxins to compare the expression level of these orthogroups between both species as mentioned above in the intraspecific variation. The toxin transcripts with expression placed out of the 99th percentile of the centered log-ratio transformed distribution of nontoxins were considered outliers that may represent differentially expressed toxins between the species.

We also performed phylogenetic inference for the main toxins groups (i.e., PLA2, SVMP, SVSP, and CTL) to better understand their evolutionary relationships. We aligned peptide sequences using MAFFT (v7.450; [57]) and used IQTree (v1.6.12; [58]) to search for the maximum likelihood tree. The final tree for each toxin family was adjusted using FigTree (v1.4.4; https://github.com/rambaut/figtree/, accessed on 25 January 2022). We then identified and mapped species-specific putative loss and duplication events onto the trees based on the phylogenetic relationships and the output of OrthoFinder. Considering that the lack of a genome does not allow a reliable annotation of such events, we marked each event as a putative duplication or loss within a species.

We performed a divergence analysis of toxin and nontoxin orthologs by calculating pairwise synonymous substitution rates (dS), nonsynonymous substitution rates (dN), and their dN/dS ratios (ω). Each orthologous pair was aligned by codon using PRANK (v.170427). The alignments were used as input to estimate dS, dN, and ω using codeml from paml package (v4.9). We used the dS, dN and ω to compare toxins against a background set of nontoxins [16] to identify if toxin genes present higher dS and/or dN and also to test for positive selection in toxins (i.e., higher values of ω). We removed orthologous pairs with dS < 0.001, to eliminate the possibility of excessively inflated ω values, and pairs with dS > 0.10 to eliminate putative misidentified orthologs. Statistical differences between toxins and nontoxins were tested using the Wilcoxon sign rank test.

### 4.5. Modularity Analysis

To check for coexpression modules that may be responsible for each species’ venom composition, we performed a weighted gene coexpression network analysis using the CEMiTool package in R (v1.14.1; [59]). We applied a variance stabilizing transformation (vst) and transcripts were filtered to reduce correlation between variance and gene expression. We used a beta value of 10 and pearson’s coefficient as the correlation method. To allow greater flexibility in identifying modules with correlated expressions we set the minimum module size to 1. We set each species as a “treatment” considered by the software to perform its comparative analysis.

### 4.6. Identification and Annotation of miRNAs

We trimmed adapters and low quality reads using cutadapt (v1.18; [60]) and used the trimmed and cleaned reads in the miRNA identification step. Due to the unavailability of *B. cotiara* and *B. fonsecai* genome sequences, we used two strategies to identify and annotate miRNAs. One using a reference-based method and the other using reference-free approaches. For the reference-based method, we followed the pipeline described by [61] using miRDeep2 and ShortStack tools with the *B. jararaca* genome as a reference [40]. To complement the miRNA identification procedure, we also applied two computational tools that are reference-free: mirnovo [62] with default parameters and the universal model designed for animals, and denomiR (https://github.com/pedronachtigall/denomiR, accessed on 25 January 2022) with default parameters. The denomiR pipeline is an experimental approach designed to identify miRNAs in small RNA sequencing data based on similarity search using a curated miRNA DB and the duplex of mature and complementary miRNA reads to detect putative novel miRNAs. To avoid putative false-positive miRNAs, we considered the miRNAs identified by the reference-based pipeline, which considers the stem-loop structure in the analysis, and the overlapped miRNAs identified by both reference-free approaches using an in-house Python script. For known miRNA identification, we combined the metazoan miRNA data available and curated at miRBase (release 22; www.mirbase.org/, accessed on 25 January 2022) and MirGeneDB (v2.0; https://mirgenedb.org/, accessed on 25 January 2022).

### 4.7. UTR Annotation and miRNA-Target Prediction

To perform miRNA-target prediction, we first annotated the 3’UTR of toxin transcripts using UTRan (https://github.com/pedronachtigall/UTRan, accessed on 25 January 2022) with default parameters. UTRan is an experimental approach designed to retrieve UTR sequences from de novo transcriptome assemblies based on read coverage data and the CDS annotation. To avoid false-positive 3’UTR sequences, we manually checked the annotated 3’UTR sequences by comparing them to the UTRs annotated for snake toxins in the NCBI nucleotide archive. Then, the miRNA-target prediction step was performed using the combination of results generated by miRanda (http://www.microrna.org/, accessed on 25 January 2022) and SeedVicious (v1.3; [63]) to improve the performance of the prediction analysis [64].

### 4.8. Venom Proteome

Venom proteome characterization was performed by liquid chromatography–tandem mass spectrometry (LC–MS/MS) by the University of São Paulo BIOMASS-Core Facility for Scientific Research (CEFAP-USP). Venom samples (50 μg of protein) were denatured with urea, reduced by triethylphosphine, and alkylated by iodoethanol before treatment with trypsin solution (0.2 μg/μL). The peptide concentration of each sample was quantified with the Qubit 2.0 Fluorometer (Life Technologies, Carlsbad, CA, USA). Peptide desalting was performed using reverse phase Empore C18-SD columns, 4mm/1mL (Sigma-Aldrich, St. Louis, MO, USA). The C18 column was equilibrated by 100% acetonitrile (ACN) and washed by 0.1% *v/v* trifluoroacetic acid (TFA) (in water) twice. The peptide pellet was resuspended in 0.1% *v/v* TFA and bound onto the column. The C18 column was washed by 0.1% *v/v* TFA twice and peptides were eluted by a solution containing 50% *v/v* ACN, 0.1% *v/v* TFA and 70% *v/v* ACN, 0.1% TFA. The eluate was dried in a speed vacuum. For the LC-MS/MS, peptide samples were resuspended in 0.1% FA and analyzed using an EASY-nLC system (Thermo Scientific) coupled to a LTQ-Orbitrap Velos mass spectrometer (Thermo Scientific). The peptides were loaded onto a C18 PicoFrit column (C18 PepMap, 75 μm id × 10 cm, 3.5 μm particle size, 100 Å pore size; New Objective, Ringoes, NJ, USA) and separated with a gradient from 100% mobile phase A (0.1% FA) to 34% phase B (0.1% FA, 95% ACN) over 60 min, 34–95% in 15 min and 5 min at 95% phase B at a constant flow rate of 250 nL/min. The LTQ-Orbitrap Velos was operated in positive ion mode with data-dependent acquisition. The full scan was obtained in the Orbitrap with an automatic gain control (AGC) target value of 10 × 10^6^ ions and a maximum fill time of 500 ms. Each precursor ion scan was acquired at a resolution of 60,000 FWHM in the 400–1500 *m/z* mass range. Peptide ions were fragmented by CID MS/MS using a normalized collision energy of 35. The 20 most abundant peptides were selected for MS/MS and dynamically excluded for a duration of 30 s. The raw data were processed and searched against an in-house database using the search tool Mascot (v2.4.1; Matrix Science). The database used to identify the MS/MS spectra is composed of the full-length precursor proteins derived from the annotated species consensus transcriptome described in the present study together with an *Escherichia coli* database. We validated the MS/MS-based peptide and protein identifications using Scaffold (v4.9.0; Proteome Software Inc., Portland, OR, USA). Peptide identities were accepted based on a 1.0% false discovery rate (FDR) using the Scaffold Local FDR algorithm. Peptide identifications were also required to exceed specific database search engine thresholds, such as Mascot ion scores >40.0 and/or X! Tandem –Log (E-value) scores >2.0. Protein identities were accepted based on an FDR of 1.0% and a minimum of two recognized unique peptides. The quantification of proteins was based on normalized Exclusive Unique Spectral Counts (EUSC), corresponding to the number of spectra attributed to proteotypic peptides of a given protein entry present in the database.

We also tested for correlations of transcriptomic and proteomic abundances of toxins within each species. First, we applied centered log-ratio (clr) transformation in the transcriptome and proteome data, which preserves rank and is equivalent to a log transformation for linear relationships [65]. This transformation was shown to allow comparisons between venom gland transcriptomes and venom proteomes [18,33]. Then, we calculated Spearman’s rank correlation coefficient, Pearson’s correlation coefficient, and the best-fit line between transcriptomes and proteomes abundances.

### 4.9. Enzymatic Activities of PLA2, SVMP, and SVSP

SVMPs, SVSPs and PLA2 enzymatic activities were assayed using synthetic substrates according to the procedures previously standardized in our lab [66]. For SVMPs, venom samples (10 μg) were incubated with 50 μM of Fluorescence Resonance Energy Transfer (FRET) Abz-AGLA-EDDnp substrate (GenOne Biotechnologies, Rio de Janeiro, RJ, Brazil), and the enzymatic reactions were monitored in a SpectraMax^®^ M2 fluorimeter (Molecular Devices, San Jose, CA, USA) with excitation at 340 nm and emission at 415 nm, at 37 ${}^{\circ }$C in kinetic mode over 10 min with a read range of 1 min. The results were expressed in Relative Fluorescence Units-RFU/min/μg. The PLA2 activity of venom samples (20 μg) was assayed using 500 μM of the substrate 4-nitro-3-[octanoyloxy] benzoic acid (Enzo Life Sciences, New York, NY, USA) incubated for 40 min at 37 ${}^{\circ }$C and activity determined according to the absorbance at 425 nm and expressed as Absorbance/min/μg of venom. For SVSPs, the venom was incubated using 800 μM of substrate Nα-benzoyl-arginyl-p-nitroanide (L-BAPNA; Sigma-Aldrich, Darmstadt, Hesse, Germany) incubated for 40 min at 37 ${}^{\circ }$C and according to absorbance at 405 nm and expressed as Absorbance/min/μg. Differences in the enzymatic activity between both species were assessed using a One-Way ANOVA and considered statistically significant at *p*-value < 0.05.

## Data Availability

The RNA-seq and miRNA-seq data sets from the venom glands of *B. cotiara* and *B. fonsecai* samples used in the present study are available at NCBI SRA under the Biosample accesses numbers SAMN16930292, SAMN16930293, SAMN16930296, and SAMN16930297.

## Supplementary Materials

The following supporting information can be downloaded at https://www.mdpi.com/article/10.3390/toxins14040237/s1. Figure S1: Venom gland transcriptome of two individuals of *Bothrops cotiara*; Figure S2: Venom gland transcriptome of two individuals of *Bothrops fonsecai*; Figure S3: Intraspecific variation in the venom proteome of *Bothrops fonsecai*; Figure S4: Comparison between levels of transcripts (TPM) and protein abundance (EUSC) in the samples analyzed of *B. cotiara* and *B. fonsecai*; Figure S5: CTL phylogeny with known alpha and beta chain CTL homologs of several snake species; Figure S6: Schematic representation of the putative evolution of PLA2 genes in *Bothrops* species; Table S1: Venom gland transcriptome of *Bothrops cotiara*; Table S2: Venom gland transcriptome of *Bothrops fonsecai*; Table S3. Orthologous annotation between *B. cotiara* and *B. fonsecai*; Table S4: Venom proteome of *B. cotiara* and *B. fonsecai*; Table S5: Modules of co-expression detected; Table S6: Venom gland miRNome of *B. cotiara* and *B. fonsecai*; Table S7: Bonafide miRNA-target interaction detected between miRNA identified and the 3’UTR annotated for toxin isoforms in *B. cotiara* and *B. fonsecai*.

## Abbreviations

The following abbreviations are used in this manuscript:

PLA2	Phospholipase A${}_{2}$
SVMP	Snake venom metalloproteinase
SVSP	Snake venom serine proteinase
CTL	C-type lectin
BPP	Bradykinin potentiating peptide
VEGF-F	Snake venom vascular endothelial growth factor
LAO	L-amino Acid Oxidase
CRISP	Cysteine-rich secretory protein
HYAL	Hyaluronidase
NGF	Venom nerve growth factor
PLB	Phospholipase B
5NUC	Snake venom 5${}^{\prime }$ nucleotidase
DIESTER	Venom phosphodiesterase
TSS	Transcription Start Site
3${}^{\prime }$UTR	Three prime untranslated region
TPM	Transcripts per million
EUSC	Exclusive Unique Spectra Counts
TMM	Trimmed Mean of M-values
dS	Synonymous substitution rate
dN	Nonsynonymous substitution rate
ω	dN/dS ratio
ρ	Spearman’s rank correlation coefficient
*R*	Pearson’s correlation coefficient
HMM	Hidden Markov Model
