# Supplementary material for: Differences in PLA2 Constitution Distinguish the Venom of Two Endemic Brazilian Mountain Lanceheads, Bothrops cotiara and Bothrops fonsecai
Source: Toxins (Basel). 2022 Mar 25;14(4):237. doi: 10.3390/toxins14040237 (PMC9028134; doi:10.3390/toxins14040237)
Supplement: Supplementary file 1 [file toxins-14-00237-s001.zip › Supplementary_file_2.pdf]

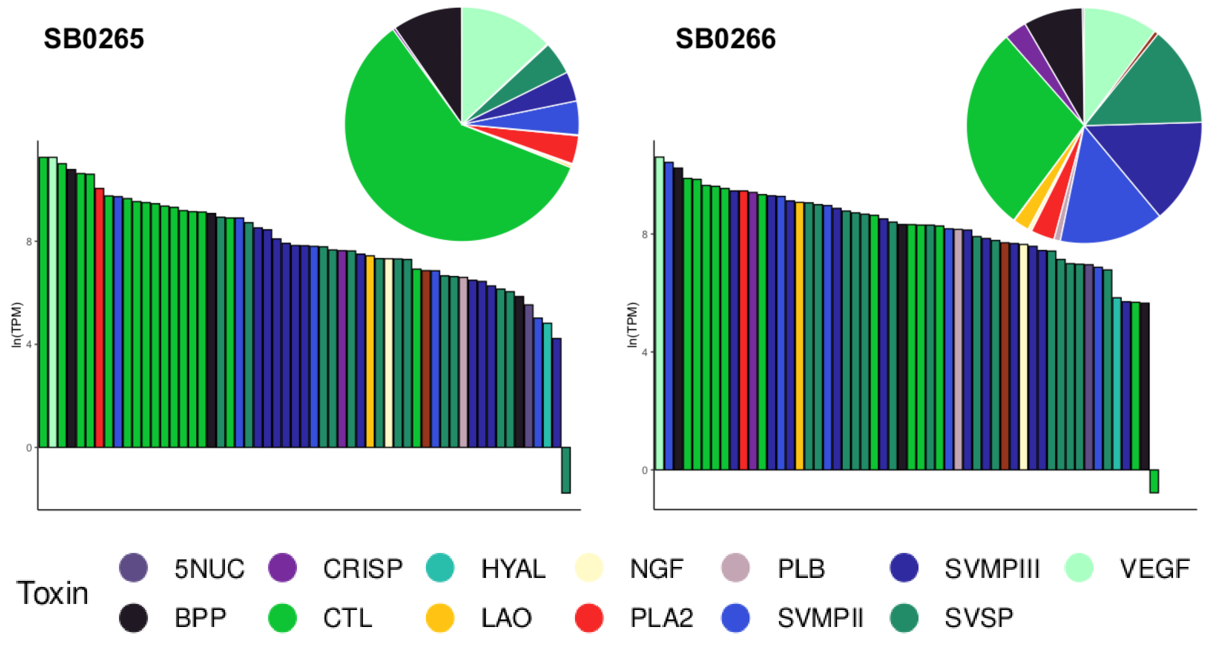

Figure S1. Venom gland transcriptome of two individuals of *Bothrops cotiara*.

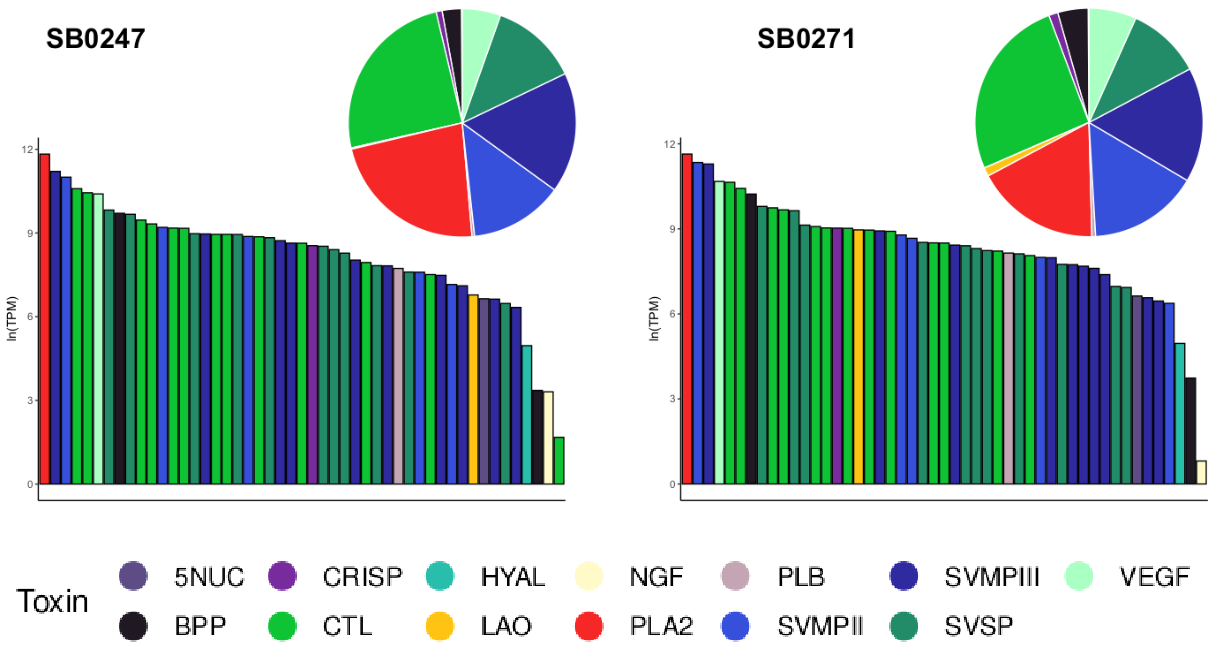

Figure S2. Venom gland transcriptome of two individuals of *Bothrops fonsecai*.

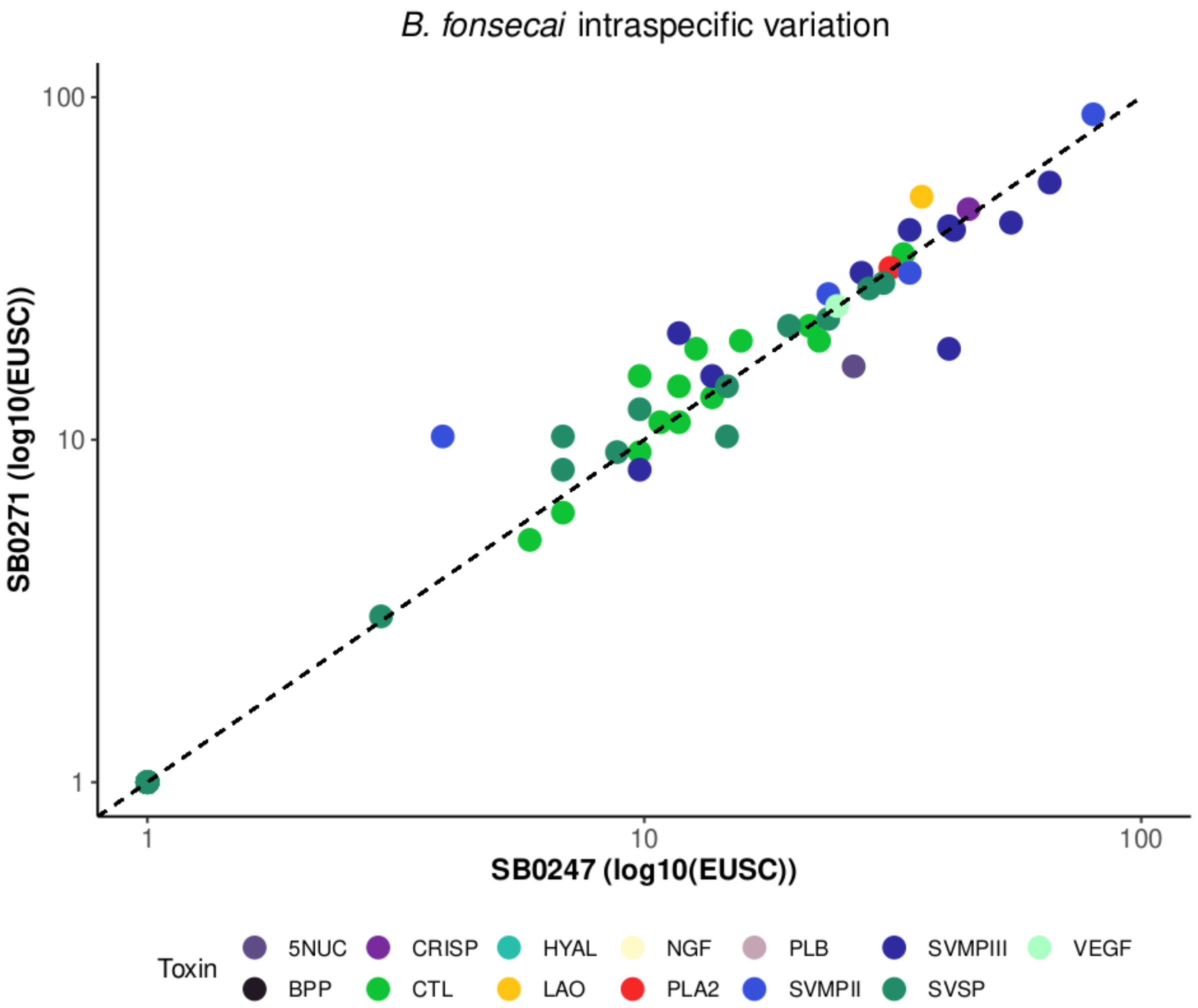

Figure S3. Intraspecific variation in the venom proteome of *Bothrops fonsecai*. The dashed line indicates a hypothetical correspondence of protein abundances between individuals.

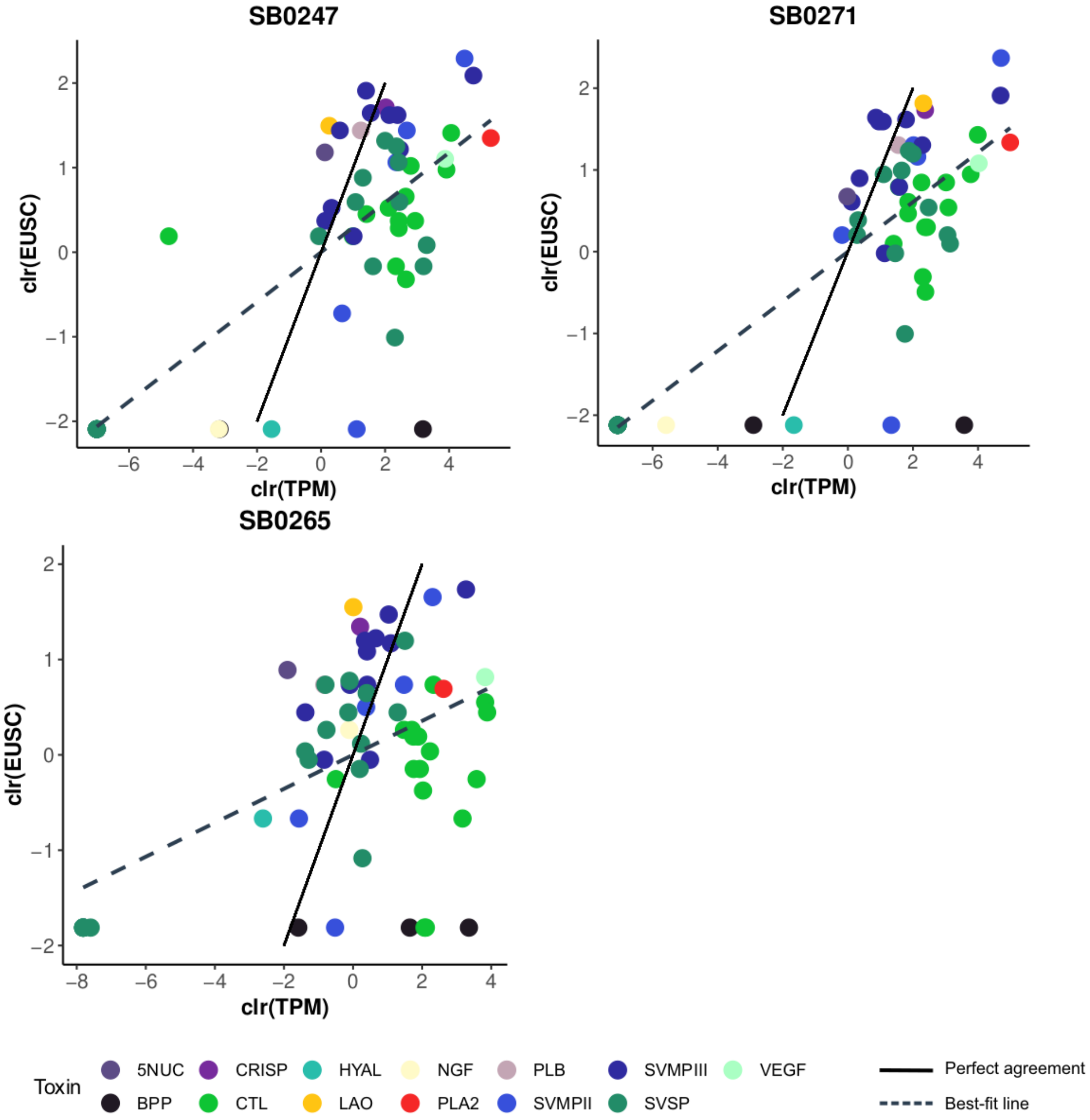

Figure S4. Comparison between levels of transcripts (TPM) and protein abundance (normalized EUSC - Exclusive Unique Spectrum Count) in the samples analyzed of *B. cotiara* (SB0265 [ρ = 0.26; *R* = 0.49]) and *B. fonsecai* (SB0247 [ρ = 0.60; *R* = 0.78] and SB0271 [ρ = 0.61; *R* = 0.81]). All data were centered log-ratio transformed (clr). Black lines indicate a perfect agreement between transcript and protein abundances. Dashed lines indicate the best-fit line between transcript and protein abundances.

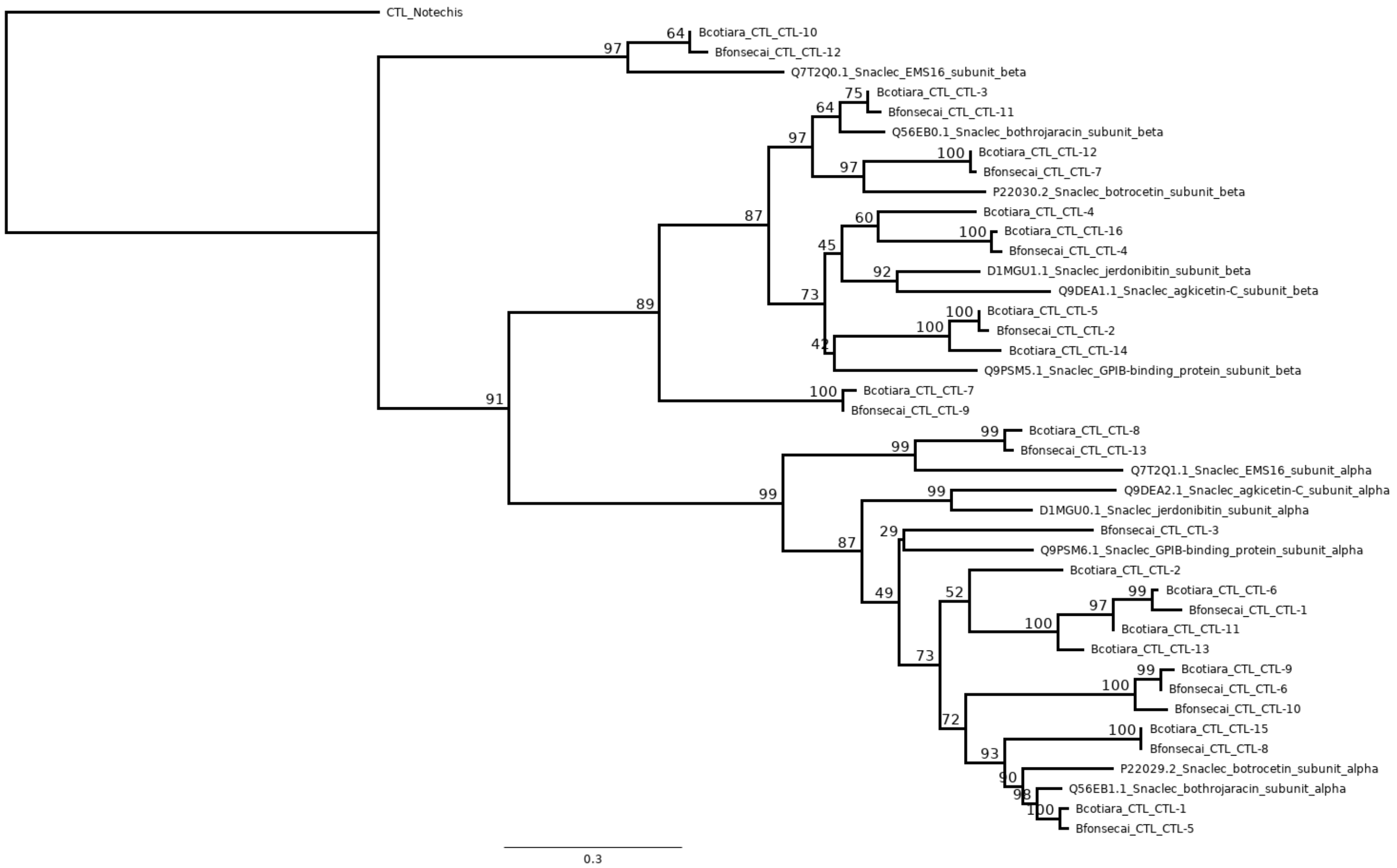

Figure S5. CTL phylogeny with known alpha and beta chain CTL homologs of several snake species. The support values of bootstrap are given in tree branches.

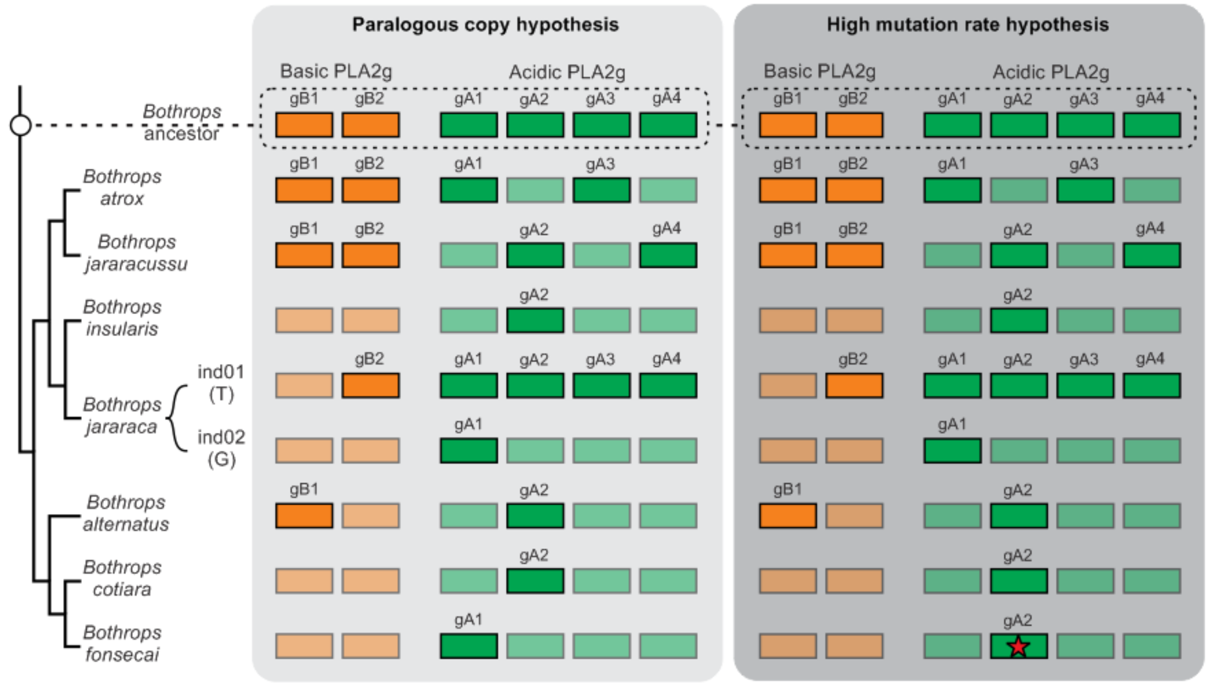

Figure S6. Schematic representation of the putative evolution of PLA2 genes in *Bothrops* species. The basic PLA2 genes are represented in orange and the acidic PLA2 are represented in green. The red star means genes with a high mutation rate. Transparent genes represent absence of genes in the species/individual. T: Transcriptome. G: Genome.
